# Statin use and the risk of herpes zoster: a nested case–control study using primary care data from the U.K. Clinical Research Practice Datalink

**DOI:** 10.1111/bjd.14815

**Published:** 2016-11-02

**Authors:** A. Matthews, M. Turkson, H. Forbes, S.M. Langan, L. Smeeth, K. Bhaskaran

**Affiliations:** ^1^ Department of Noncommunicable Diseases EpidemiologyLondon School of Hygiene and Tropical MedicineLondonU.K.

## Abstract

**Background:**

Statins are commonly prescribed worldwide and recent evidence suggests that they may increase the risk of herpes zoster (HZ).

**Objectives:**

To quantify the effect of statin exposure on the risk of HZ in the U.K.

**Methods:**

A matched case–control study was conducted using data from U.K. primary care and hospital records. Patients > 18 years with an incident diagnosis of HZ were matched to up to four controls for age, sex and general practice. Patients were included in the statin exposure group if they had ever used a statin, and the daily dosage of the most recent statin prescription and the time since the most recent statin prescription were also recorded. The primary outcome was an incident diagnosis of HZ. Odds ratios (ORs) were estimated from conditional logistic regression and adjusted for potential confounders.

**Results:**

A total of 144 959 incident cases of HZ were matched to 549 336 controls. Adjusted analysis suggested strong evidence for an increase in the risk of HZ related to statin exposure (OR 1·13, 95% confidence interval 1·11–1·15). There was also an increased risk when dosages were increased for patients who were currently or had recently been receiving statin treatment (*P*_trend_ < 0·001), and we found an attenuation of the increased risk of HZ in previous statin users as the time since last statin exposure increased (*P*_trend_ < 0·001).

**Conclusions:**

These findings are consistent with the hypothesis that statin therapy leads to an increase in the risk of HZ.

## Abstract 2

**What's already known about this topic?**

- Studies in both Canada and Taiwan have recently reported a small but significantly increased risk of herpes zoster (HZ) in patients receiving statin treatment.
- As statins are one of the most widely prescribed drugs in the U.K., with around 45 million prescriptions every year, any adverse effects will have substantial public health implications.

**What does this study add?**

- In this large matched case–control study, statin exposure was associated with a modest increase in the risk of HZ.
- A dose–response relationship was observed, and there was an attenuation of the increased risk over time among people who stopped statin therapy, indicating that the increased risk is consistent with a causal effect.
- There may be extra motivation to maximize HZ vaccine uptake among eligible patients receiving a statin.

**Linked Comment:** Shalom and Cohen. *Br J Dermatol* 2016; **175**:1137–1138.

Herpes zoster (HZ), commonly known as shingles, is caused by the reactivation of latent varicella zoster virus when specific cell‐mediated immunity becomes compromised. HZ presents as a painful dermatomal vesicular rash. Healing occurs over a period of 2–4 weeks and often results in scarring and permanent localized changes in skin pigmentation.^1^ The incidence of HZ is strongly associated with age, and 30% of cases occur in patients aged over 55 years.^2^ A range of conditions such as rheumatoid arthritis, inflammatory bowel disease, chronic obstructive pulmonary disorder, chronic kidney disease and depression are also associated with an increased risk of HZ.^3^ Postherpetic neuralgia (PHN) develops in 12% of patients with HZ aged 50 years or older^1^, ^4^ and may be associated with intense pain, which can last for years. A live HZ vaccine is available with efficacy in reducing the risk of HZ and PHN among immunocompetent patients over 50 years old,^5^, ^6^, ^7^ although the vaccine is routinely available only for patients aged 70–79 years in the U.K.^8^ and is recommended for patients aged over 60 years in the U.S.A.

Statins are lipid‐lowering drugs that reduce the risk of cardiovascular disease (CVD) in both primary and secondary care.^9^, ^10^, ^11^, ^12^ In the 12 months preceding March 2008, 45·2 million statin prescriptions were dispensed in primary care in England making them the most commonly prescribed class of drugs in the U.K.^13^ In addition to being lipid‐lowering agents, it has been posited that statins may also modulate systemic immune responses.^14^ Antoniou *et al*.^15^ and Chen *et al*.^16^ recently reported a small but significantly increased risk of HZ among patients from Ontario, Canada [hazard ratio (HR) 1·13, 95% confidence interval (CI) 1·10–1·17] and Taiwan (HR 1·21, 95% CI 1·13–1·29), respectively. Although the mechanisms by which statins may increase the risk of HZ are not established, it has been hypothesized that statins have immunomodulating properties that operate by decreasing the synthesis of isoprenoid phosphates, which are required for the activation of Ras‐related GTPases,^17^ causing the impairment of T‐cell activation and proliferation.

As statins are commonly prescribed worldwide, any adverse effects may have substantial public health implications. An increased risk of HZ would not only have an impact on the quality of life of affected patients, but could also add to the burden on health services, given the high cost of treatment for PHN.^18^ Hence, this study aims to quantify the effect of statin exposure on the risk of HZ in the general population of the U.K.

## Patients and methods

A matched case–control study was conducted to quantify the effects of statin use on the risk of HZ in the general population of the U.K.

### Data source

The data source for this study was the U.K. Clinical Practice Research Datalink (CPRD), which is a primary case database comprising information from general practitioners (GPs) who use the Vision IT system and who have agreed to participate at the practice level.^19^ The CPRD contains anonymized primary care data from approximately 9% of the U.K. population and is broadly representative of the characteristics of patients and practices in the U.K.^20^ Overall, 60% of patients in the CPRD have linked data available in the Hospital Episode Statistics (HES), which has recorded hospital attendances in England since 1997.

### Selection of cases

Patients with HZ were identified from the CPRD and linked HES data. All patients in the study population were ≥ 18 years and were followed‐up at any time between 1 January 2000 and 31 December 2011. Patients were classified as cases in the CPRD if they had a first ever HZ diagnosis recorded during the study period and at least 12 months of follow‐up in the CPRD prior to this first diagnosis of HZ. The 12‐month restriction was intended to exclude previous cases of HZ that were retrospectively recorded soon after registration at a general practice.^21^ In HES, incident HZ was identified according to International Classification of Diseases 10th revision codes (B02, B02·0, B02·1, B02·31, B02·7, B02·8, B02·9 and G53·0) that appeared in the primary diagnosis field and the index date was the hospital admission date of the first episode. The earliest record of HZ was used if HZ was recorded in both HES and the CPRD for the same patient.

### Selection of controls

Up to four control patients were selected for each HZ case by incidence density sampling, matched for GP practice, age (within 1 year) and sex, and without reference to statin exposure status. Controls had to be registered with no history of HZ or PHN on the index date of their matched case, and have at least 12 months of previous follow‐up in the CPRD prior to this date. When incidence density sampling is used, the odds ratio (OR) obtained from a case–control study unbiasedly estimates the rate ratio in the study base.^22^ Matching for practice was done to minimize confounding as a result of differences in GP practice policies and procedures, and also to minimize confounding by factors associated with geographical area, including socioeconomic status. The index date for the controls was set to that of their matched case. A control patient could also later be included as a case if they developed HZ after this date. Potential controls were assumed to be inactive with the practice and were excluded if they had no contact with their GP practice at any time between 6 months before and 12 months after the index date.

### Exposure

The primary exposures were (i) ever having been exposed to a statin; and (ii) time since last exposure to a statin. The most recent prescription of a statin before the index date was initially identified and patients were then categorized as having ever been exposed to a statin or having never been exposed to a statin.

To calculate the time since last exposure, the duration of the most recent prescription before the index date was calculated based on the number of tablets prescribed, combined with daily dosing instructions. When the number of tablets or dosing instructions were not provided, the median number of tablets prescribed and the number of tablets to be taken each day were imputed. Next, 30 days were added to the prescription end date as a grace period to indicate that a patient could still be taking the same pills during this time period owing to an excess of medication. Current statin use was defined as having a prescription for which the calculated duration included the index date. The number of months since statin exposure was calculated for noncurrent users by calculating the time between the end of the latest prescription plus the 30‐day grace period and the index date. ‘Months since exposure’ was assessed using the following categories: current, 0–12 months since exposure, 12–36 months since exposure and > 36 months since exposure.

As a secondary exposure, the daily dosage of the most recent statin prescription was stratified into one of three categories based on published estimates of expected reductions in low‐density lipoprotein cholesterol from baseline.^11^ These categories were low (atorvastatin < 20 mg, rosuvastatin < 10 mg, cerivastatin < 0·3 mg, simvastatin < 80 mg, fluvastatin at all dosages, pravastatin at all dosages, lovastatin at all dosages), medium (atorvastatin 20 mg to < 80 mg, rosuvastatin 10 mg to < 40 mg, simvastatin ≥ 80 mg, cerivastatin 0·3 mg to 0·4 mg) and high (atorvastatin ≥ 80 mg, rosuvastatin ≥ 40 mg, cerivastatin ≥ 0·4 mg).

As a further exposure, ‘duration of continuous use’ was calculated by retrospectively accumulating each patient's prescriptions until there was a gap between the date of prescription and the end of the previous prescription. The date of the earliest prescription was then compared with the end date of the latest prescription for noncurrent statin users, and the date of the earliest prescription was compared with the index date for current statin users. The cumulative length of the most recent exposure was categorized as < 12 months or > 12 months.

### Statistical analysis

#### Primary analysis

The characteristics of the study population were described according to case–control status. Conditional logistic regression was then used for analysis, so all ORs accounted for the matched variables of age (within 1 year), sex, practice and calendar time. Univariate ORs, with 95% CIs, were initially calculated to explore the association between the risk of HZ and primary exposures of ‘ever exposed to a statin’ (ever/never exposed, regardless of timing), and ‘time since last exposure’ (current, 0–12 months since exposure, 12–36 months since exposure and > 36 months since last statin exposure). Multivariate analyses were then carried out including the following possible HZ risk factors: body mass index (BMI) category [underweight (< 18·5 kg m^−2^), normal weight (18·5–24·9 kg m^−2^), overweight (≥ 25–29·9 kg m^−2^), obese (≥ 30 kg m^−2^)], smoking status, alcohol use, CVD, HIV, lymphoma, leukaemia, myeloma, haematopoietic stem cell transplantation, other immunosuppressive therapy, other unspecified cellular immune deficiencies, oral corticosteroids, rheumatoid arthritis, systemic lupus erythematosus, chronic obstructive pulmonary disorder, asthma, chronic kidney disease, depression, cancer and diabetes.^3^ Details of how these risk factors were defined are outlined in Appendix 1. All ORs were calculated using the baseline group of patients who had never been exposed to statins. A complete case analysis was performed, such that individuals with missing data for BMI, smoking or alcohol were excluded; this relies on the assumption that the probability of these data being missing is independent of HZ risk, conditional on covariates.^23^

#### Secondary analysis

Adjusted ORs, with 95% CIs, were calculated to explore the association between the risk of HZ and the dosage of the latest prescription (low, medium, high), stratified by time since last exposure. For this analysis, owing to the low number of patients, patients in the 12–36 months and > 36 months since last prescription groups were collated. The association between the risk of HZ and the duration of continuous use (0–12 months, > 12 months), stratified by time since last exposure, was also explored.

### Effect modification

A potentially effect‐modifying role of age at diagnosis (index) date was explored using the likelihood ratio test and by calculating stratum‐specific ORs in the multivariable model. For this analysis, age was treated as a binary variable, using a cut‐off point of 70 years with the rationale that these results might inform HZ vaccine policy and the fact that the HZ vaccine is not routinely available for patients under the age of 70 years in the U.K.

### Sensitivity analyses

To assess the presence of ascertainment bias, exposure to angiotensin‐converting enzyme (ACE) inhibitors was used as a negative control as they are prescribed with similar regularity and duration as statins and there is no known association between these drugs and HZ. To assess the possibility of exposure misclassification in primary analyses, we carried out an analysis where a patient was classified as exposed to statins only if they had been continuously prescribed statins for at least 3 months. Furthermore, to assess the short‐term effects of statins on the risk of HZ, we further stratified the time since last exposure analysis to include ≤ 3 months since a statin prescription (new categories: current, ≤ 3 months since stopping statins, > 3–12 months since stopping statins, > 12–36 months since stopping statins, > 36 months since stopping statins).

## Results

### Descriptive analysis

A total of 144 959 incident cases of HZ were identified. They were matched to 549 336 controls who were not diagnosed with HZ. Table 1 outlines the descriptive details of the cases and controls. Overall, 59·4% of cases and 61·0% of controls were female. A total of 22·2% of cases and 20·2% controls had ever been prescribed a statin prior to index date. We found that 14·2% of cases and 16·1% of controls had at least one variable with missing data (either alcohol status, BMI category, smoking status or a combination of all three variables).

**Table 1:** Description of cases and controls

Matching factors	Cases (*N* = 144 959)	Controls (*N* = 549 336)
Ever prescribed a statin		
Yes	32 119 (22·2)	111 023 (20·2)
No	112 840 (77·8)	438 313 (79·8)
Sex		
Male	58 888 (40·6)	214 064 (39·0)
Female	86 071 (59·4)	335 272 (61·0)
Age		
18–29	10 849 (7·48)	38 761 (7·06)
30–49	28 762 (19·84)	104 708 (19·06)
50–59	27 833 (19·2)	105 157 (19·1)
60–69	31 134 (21·5)	121 108 (22·0)
70–79	28 025 (19·3)	110 097 (20·0)
80–89	15 891 (11·0)	61 566 (11·2)
≥ 90	2465 (1·7)	7939 (1·4)
Socioeconomic status (practice level)		
1	28 938 (20·0)	109 663 (20·0)
2	28 853 (19·9)	109 253 (19·9)
3	29 811 (20·6)	112 888 (20·5)
4	30 550 (21·1)	115 678 (21·1)
5	26 807 (18·5)	101 854 (18·5)
Other characteristics		
Mean (IQR) length of follow‐up, years	8·6 (4·3–12·1)	8·6 (4·3–12·1)
Body mass index category		
Underweight	2776 (1·9)	10 549 (1·9)
Normal weight	50 530 (34·9)	188 060 (34·2)
Overweight	47 886 (33·0)	177 603 (32·3)
Obese	29 581 (20·4)	109 440 (19·9)
Missing	14 186 (9·8)	63 684 (11·6)
Smoking status		
Nonsmoker	54 751 (37·8)	208 436 (37·9)
Current smoker	36 107 (24·9)	141826 (25·8)
Former smoker	52 353 (36·1)	186 373 (33·9)
Missing	1784 (1·2)	12 701 (2·3)
Alcohol use		
Nondrinker	14 481 (10·0)	56 774 (10·3)
Current drinker	103 113 (71·1)	383 976 (69·9)
Former drinker	12 786 (8·8)	45 242 (8·2)
Missing	14 579 (10·1)	63 344 (11·5)
Cardiovascular disease	15 855 (10·94)	53 352 (9·71)
HIV	128 (0·09)	97 (0·02)
Lymphoma	444 (0·31)	386 (0·07)
Leukaemia	205 (0·14)	368 (0·07)
Myeloma	492 (0·34)	816 (0·15)
Haematopoietic stem cell transplantation	26 (0·02)	3 (0·00)
Other unspecified cellular immune deficiencies	95 (0·07)	190 (0·03)
Other immunosuppressive therapy	2164 (1·49)	3822 (0·70)
Oral corticosteroids	502 (0·35)	1058 (0·19)
Rheumatoid arthritis	3111 (2·15)	8029 (1·46)
Systemic lupus erythematosus	387 (0·27)	818 (0·15)
Inflammatory bowel disease	1851 (1·28)	5118 (0·93)
COPD	6815 (4·70)	20 201 (3·68)
Asthma	10 243 (7·07)	31 865 (5·80)
Chronic kidney disease	8724 (6·02)	29 437 (5·36)
Depression	6830 (4·71)	22 052 (4·01)
Diabetes	11 430 (7·88)	41 320 (7·52)
Type 1	396 (0·27)	1054 (0·19)
Type 2	10 359 (7·15)	38 136 (6·94)
Unknown	675 (0·47)	2130 (0·39)
Missing data in any variable	20 598 (14·2)	88 233 (16·1)

IQR, interquartile range; COPD, chronic obstructive pulmonary disease. Values are *n* (%) unless otherwise stated.

### Statin exposure

Univariate analysis accounting for the matched variables age, sex and GP practice, presented strong evidence for an increase in risk of HZ associated with ever having been exposed to a statin (OR 1·20, 95% CI 1·18–1·22). This association was attenuated when fully adjusted for potential confounders (OR 1·13, 95% CI 1·11–1·15), but there was still strong evidence to suggest a modest increase in the risk of HZ associated with ever having been exposed to a statin.

### Time since last exposure

**Figure 1:**
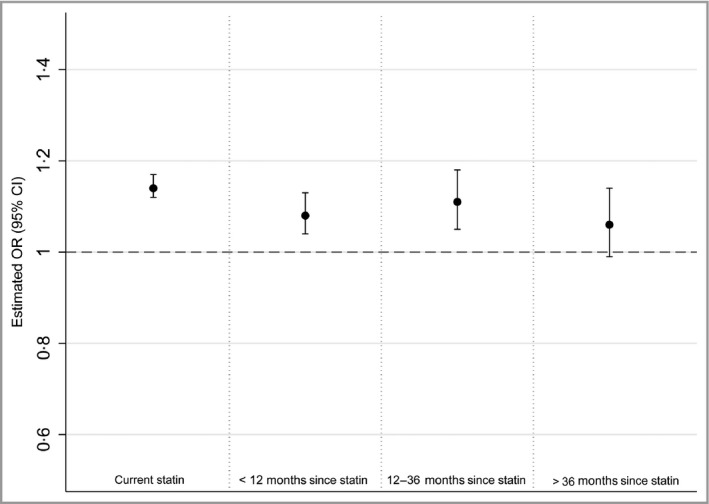
Adjusted odds ratio (OR) for association between time since end of last statin prescription and HZ. The reference category for all estimations is patients who have never been prescribed a statin. All ORs are adjusted for body mass index category, smoking status, alcohol use, cardiovascular disease, lymphoma, leukaemia, myeloma, haematopoietic stem cell transplantation, other immunosuppressive therapy, other unspecified cellular immune deficiencies, oral corticosteroids, rheumatoid arthritis, systemic lupus erythematosus, chronic obstructive pulmonary disease, asthma, chronic kidney disease, depression, cancer and diabetes. CI, confidence interval.

Table 2 and Figure 1 show unadjusted and adjusted ORs for the effect of statin use on the risk of developing HZ according to the timing of the last exposure to statins. There was strong evidence of an attenuation of the increased risk of HZ associated with statin use as the time since the last prescription increased (*P*_trend_ < 0·001).

**Table 2:** Odds ratios for the association between both ever having been exposed to a statin plus time since last statin and herpes zoster

Statin use	Odds ratio (95% CI)		
	Model 1	Model 2	Model 3
Never	1·00	1·00	1·00
Ever	1·20 (1·18–1·22)	1·17 (1·15–1·19)	1·13 (1·11–1·15)
Current	1·21 (1·19–1·23)	1·18 (1·15–1·20)	1·14 (1·12–1·17)
< 12 months since stopping statins	1·15 (1·11–1·20)	1·12 (1·07–1·17)	1·08 (1·04–1·13)
12–36 months since stopping statins	1·17 (1·11–1·23)	1·15 (1·08–1·21)	1·11 (1·05–1·18)
> 36 months since stopping statins	1·13 (1·06–1·21)	1·09 (1·02–1·17)	1·06 (0·99–1·14)

CI, confidence interval. Model 1: unadjusted model. Model 2: unadjusted model, restricted to patients who had no missing data in all descriptive variables. Model 3: Adjusted for body mass index category, smoking status, alcohol use, cardiovascular disease, HIV, lymphoma, leukaemia, myeloma, haematopoietic stem cell transplantation, other immunosuppressive therapy, other unspecified cellular immune deficiencies, oral corticosteroids, rheumatoid arthritis, systemic lupus erythematosus, chronic obstructive pulmonary disease, asthma, chronic kidney disease, depression, cancer and diabetes.

### Dosage

**Figure 2:**
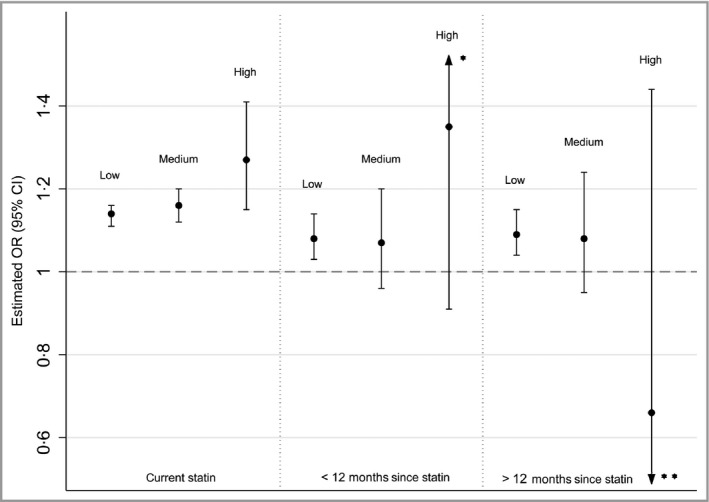
Adjusted odds ratio (OR) for association between dosage of last statin prescription and herpes zoster, stratified by time since last statin prescription. The reference category for all estimations is patients who have never been prescribed a statin. All ORs are adjusted for body mass index category, smoking status, alcohol use, cardiovascular disease, lymphoma, leukaemia, myeloma, haematopoietic stem cell transplantation, other immunosuppressive therapy, other unspecified cellular immune deficiencies, oral corticosteroids, rheumatoid arthritis, systemic lupus erythematosus, chronic obstructive pulmonary disease, asthma, chronic kidney disease, depression, cancer and diabetes. *Upper confidence interval (CI) = 2·01. **Lower CI = 0·30.

Table 3 and Figure 2 show unadjusted and adjusted ORs for the effect of the dosage of the most recent statin prescription and the risk of HZ, stratified by time since last exposure. Among current statin users and recent statin users (those with a last prescription < 12 months before index date), there was strong evidence for an increasing trend in the risk of HZ as statin dosage increased (*P*_trend_ < 0·001 in both instances). CIs were very wide for individuals who stopped their statin therapy > 12 months before the index date.

**Table 3:** Odds ratios for the association between dosage of last statin prescription and herpes zoster, stratified by time since last exposure

Statin use	Odds ratio (95% CI)		
	Model 1	Model 2	Model 3
Never	1·00	1·00	1·00
Current			
Low	1·20 (1·18–1·23)	1·17 (1·14–1·19)	1·14 (1·11–1·16)
Medium	1·23 (1·19–1·27)	1·20 (1·16–1·24)	1·16 (1·12–1·20)
High	1·40 (1·27–1·54)	1·35 (1·22–1·50)	1·27 (1·15–1·41)
< 12 months since stopping statins			
Low	1·14 (1·09–1·20)	1·12 (1·07–1·18)	1·08 (1·03–1·14)
Medium	1·18 (1·06–1·31)	1·12 (1·00–1·25)	1·07 (0·96–1·20)
High	1·49 (1·03–2·16)	1·40 (0·94–2·07)	1·35 (0·91–2·01)
> 12 months since stopping statins			
Low	1·15 (1·10–1·21)	1·13 (1·07–1·18)	1·09 (1·04–1·15)
Medium	1·14 (1·00–1·29)	1·12 (0·98–1·28)	1·08 (0·95–1·24)
High	0·93 (0·47–1·85)	0·70 (0·33–1·51)	0·66 (0·30–1·44)

CI, confidence interval. Model 1: unadjusted model. Model 2: unadjusted model, restricted to patients who had no missing data in all descriptive variables. Model 3: adjusted for body mass index category, smoking status, alcohol use, HIV, cardiovascular disease, lymphoma, leukaemia, myeloma, haematopoietic stem cell transplantation, other immunosuppressive therapy, other unspecified cellular immune deficiencies, oral corticosteroids, rheumatoid arthritis, systemic lupus erythematosus, chronic obstructive pulmonary disease, asthma, chronic kidney disease, depression, cancer and diabetes.

### Duration of continuous use

The results from fully adjusted analyses for duration of continuous use, stratified by the time since last exposure, showed no clear evidence that the length of statin prescription modified the effect of statins on the risk of HZ. Full results are shown in Appendices 2 and 3.

### Effect modification

There was no evidence that age (treated as a binary variable indicating whether a patient was aged over 70 years) modified the effect of statins on the risk of HZ [*P* = 0·41 (stratified results in Appendix 4)].

### Sensitivity analyses

#### Negative control

When fully adjusted for all covariates, there was evidence of a small increase in the risk of HZ associated with ever having been exposed to an ACE inhibitor compared with patients who had never been exposed to an ACE inhibitor (OR 1·03, 95% CI 1·01–1·05; Table 4).

**Table 4:** Odds ratio for the association between ever having been exposed to an angiotensin‐converting enzyme (ACE) inhibitor and herpes zoster

ACE inhibitor use	Odds ratio (95% CI)		
	Model 1	Model 2	Model 3
Never	1·00	1·00	1·00
Ever	1·11 (1·10–1·13)	1·09 (1·07–1·04)	1·03 (1·01–1·05)

CI, confidence interval. Model 1: unadjusted model. Model 2: unadjusted model, restricted to patients who had no missing data in all descriptive variables. Model 3: adjusted for body mass index category, smoking status, alcohol use, cardiovascular disease, HIV, lymphoma, leukaemia, myeloma, haematopoietic stem cell transplantation, other immunosuppressive therapy, other unspecified cellular immune deficiencies, oral corticosteroids, rheumatoid arthritis, systemic lupus erythematosus, chronic obstructive pulmonary disease, asthma, chronic kidney disease, depression, cancer and diabetes.

#### Statin prescriptions of at least 3 months

When the patients who had been exposed to statins were required to have at least 3 months of statin prescriptions before being categorized into the exposure group, there was still evidence to suggest that ever having been exposed to statins increased the risk of HZ (OR 1·12, 95% CI 1·09–1·14) when adjusted for all potential confounders.

#### Time since last exposure (short‐term effects)

Appendix 5 shows unadjusted and adjusted ORs for the effect of statin use on the risk of developing HZ according to the time of the last exposure to statins, including a category estimating the risk in patients whose last exposure was ≤ 3 months prior to the index date. There continued to be strong evidence of an attenuation of the increased risk of HZ associated with statin use as the time since last prescription increased (*P*_trend_ < 0·001).

## Discussion

In this large matched case–control study, statin exposure was associated with a modest increase in the risk of HZ (OR 1·13, 95% CI 1·11–1·15). A dose–response relationship was observed and there was an attenuation of the excess risk over time among people who had stopped their statin therapy. These observations are consistent with a causal effect. An additional analysis was conducted using ACE inhibitors as a negative control exposure and we witnessed some association, suggesting that a small proportion of the observed association between statins and HZ may be attributable to ascertainment bias or confounding by indication, but the effect size (OR 1·03) suggests that this is unlikely to provide a comprehensive explanation of the association for statins.

All incident cases of HZ were identified in this large population‐based dataset during the 11‐year follow‐up period, hence this study was highly powered to detect small effect sizes for the association between the use of statins and the risk of HZ.

The study may be prone to misclassification of HZ because primary care diagnosis of HZ in the U.K. is clinically based with no laboratory testing available. However, a validation study in the Netherlands found that over 90·8% of diagnosed cases in general practice had antibodies indicating recent HZ infection, suggesting that a clinical HZ diagnosis has a high positive predictive value.^24^

A complete case analysis was used, which relies on the assumption that the probability of these data being missing is independent of HZ risk, conditional on covariates.^23^ Given that only 14·2% of cases and 16·1% of controls had missing data regarding smoking status, alcohol usage or BMI category, any violation of this assumption is unlikely to have a significant effect on the results. Furthermore, in unadjusted analyses, effect estimates restricted to patients with no missing data were similar to estimates for the full study population.

There would be a risk of residual confounding by indication if the indication for statins also increased the risk of HZ. Statins are prescribed for both primary and secondary prevention of CVD, which is not known to be an important risk factor for HZ. All multivariate models were adjusted for prior CVD, and this adjustment had little impact on the estimated effect of statins on HZ risk, suggesting minimal confounding. It was not possible to adjust directly for confounding by indication in primary prevention (i.e. a high risk of CVD, without an actual prior CVD diagnosis), although we did adjust for important drivers of high CVD risk including age, sex, BMI, smoking, diabetes, chronic kidney disease, cancer and rheumatoid arthritis. However, some residual confounding, for example by cholesterol level,^25^ cannot be ruled out.

We were unable to obtain a reliable estimate of the adherence to statins for the patients included in the study, and therefore were unable to assess the association between adherence to statin therapy and the risk of developing HZ. This was because CPRD does not collect data on drug adherence, and there was a substantial amount of missing information in the data required to estimate prescription duration, making it difficult to estimate adherence indirectly.

In our negative control analysis, we observed some association between the use of ACE inhibitors and HZ risk. The effect size was substantially smaller than in our main analysis, and the statistical significance should not be overemphasized given the very high statistical power available. Nevertheless, the analysis suggests that a small part of the estimated association for statins may have been driven by noncausal factors; ascertainment bias and confounding by indication being the two most likely contenders. Ascertainment bias would occur if patients receiving regular preventative medications, such as statins or ACE inhibitors, were more likely to have an HZ diagnosis recorded as a result of the greater amount of GP contact associated with using these medications. We believe that ascertainment bias is likely to have a limited impact as HZ frequently presents with a very painful rash. A survey regarding immunization practices in the U.S. among people aged 60 years and older found that 95% of those who knew they had HZ sought care from their GP, hence the majority of patients with HZ consult their GP regardless of other GP contact. In the U.K. context, where healthcare is free at the point of delivery, we would expect that almost all patients would attend for care if they develop HZ. Residual confounding by indication may be a more likely explanation of this association. As discussed above, we adjusted for prior CVD and for important drivers of CVD, but given the limitations of the data, some residual confounding is possible, and would likely apply similarly to both the ACE inhibitors and statins analyses, as the two drug classes have related indications.

We carried out two sensitivity analyses to assess potential biases within the analysis strategy. The first analysis consisted of requiring at least 3 months of statin prescriptions before defining a patient as being exposed to statins. Imposing this restriction reduced the effect size but not enough to change the final conclusions, which shows that the original criteria for exposure were adequate to avoid exposure misclassification. We also further stratified the time since last exposure analysis to include a category assessing the risk in patients whose last exposure was ≤ 3 months prior to the index date. We continued to observe an attenuation of the risk of HZ as time since exposure increased, with a relatively higher risk in those with the most recent prescriptions.

Although Antoniou *et al*.^15^ and Chen *et al*.^16^ undertook similar studies in Ontario, Canada and Taiwan, respectively, this is the first study of its type within a U.K. setting. The strength of the association between the risk of HZ and statin use is consistent with that found in both previous studies (HR 1·13, 95% CI 1·10–1·17 and HR 1·21, 95% CI 1·13–1·29, respectively). However, neither of these studies attempted to explore the association between time since last statin use and HZ infection. Our study found that HZ risk decreased as the time since last statin exposure increased, suggesting that the risk of HZ can be reduced if the patient stops statin therapy.

In this study, a dose–response relationship was found in current statin users and although Antoniou *et al*. classified statin dosage in the same manner as this study, they found no dose–response relationship. However, their study was restricted to patients over the age of 66 years, whereas this study included all patients with HZ and matched controls over the age of 18 years. Chen *et al*. reported a higher risk of HZ associated with all statins prescribed at high daily dosages in comparison with those prescribed at low daily dosages. They stated that high dosages of statins are prescribed to patients with high cholesterol levels, and there is limited evidence suggesting that high cholesterol levels are associated with HZ,^25^ which could partly explain the association found between statins and HZ.

In conclusion, this study adds to the growing literature suggesting that statin therapy may lead to a modest increase in the risk of HZ. It is clear that the preventive benefits of statin therapy are likely to outweigh the limited increase in HZ risk in many cases. However, this evidence should be taken into account by GPs when prescribing statins to those at high risk of HZ. We would also suggest that there may be an extra motivation to maximize HZ vaccine uptake among eligible patients who are also receiving a statin.

## Supporting information


**Video S1**. Author video.

## Appendix 1

### Further detail on defining risk factors

- Rheumatoid arthritis and systemic lupus erythematosus were defined as a diagnosis prior to the index date
- Patients with chronic obstructive pulmonary disorder were defined as those with a diagnosis of chronic obstructive pulmonary disorder, including chronic bronchitis and emphysema, prior to the index date and ≥ 35 years at first chronic obstructive pulmonary disorder diagnosis. Patients with asthma were those with an asthma diagnosis before the index date and an asthma‐related prescription (short‐ and long‐acting beta‐2 agonists and antimuscarinics, inhaled corticosteroids, cromoglycates and nedocromil, theophyllines, leukotriene receptor agonists and omalizumab within 12 months prior to the index date; patients with a chronic obstructive pulmonary disorder diagnosis in their medical history were not classified as asthmatic)
- Cardiovascular disease was defined as a patient having a diagnosis of any cardiovascular‐related disease (myocardial infarction, angina, revascularisation procedures, stroke, transient ischaemic attack, abdominal aortic aneurism or intermittent claudication) before the index date
- Patients with chronic kidney disease were those with a diagnosis of mild, moderate or severe chronic kidney disease, kidney transplant or kidney dialysis, at any time prior to the index date
- Depression was defined as having a diagnosis or symptom of depression (such as ‘feeling depressed’ or ‘sad mood’) or within 1 year prior to the index date
- To define diabetes, a definite diabetes diagnosis, or a possible diabetes code (e.g. self‐ monitoring of blood glucose) with a subsequent diabetes‐specific prescription (insulin or oral antidiabetics) or two or more diabetes drug prescriptions was required prior to the index date; gestational diabetes and drug‐induced diabetes were excluded. Age at first diagnosis, age at first treatment and treatment received to classify patients into type 1 or type 2 diabetes were also included. Patients were categorized as type 1 or type 2 diabetes where possible. Distinguishing between type 1 and type 2 diabetes was not always possible from diabetes codes as patients are frequently given a nonspecific code. Therefore we chose not to use this information, but rather used age at first diagnosis, age at first treatment and treatment received to classify diabetes type, as in previous Clinical Practice Research Datalink studies. Type 1 was assigned where age at first diagnosis was ≤ 35 years and treatment was exclusively insulin or where patients received at least two insulin prescriptions ≤ 35 years, but had no diabetes diagnosis. Type 2 was assigned where age at first diabetes diagnosis was > 35 years or where patients received exclusively oral antidiabetics at > 35 years of age. Patients with age at diagnoses > 35 years but treated exclusively with insulin and those not fitting into these categories were assigned as ‘unknown type’
- Severely immunosuppressive conditions determined by the Advisory Committee on Immunization Practices to be contraindications to vaccination included were, recent history (less than 2 years before index date) of leukaemia, or any history of HIV, haematopoietic stem cell transplantation, myeloma or ‘other unspecified cellular immune deficiencies’ (e.g. pancytopenia)
- For immunosuppressive treatments, all relevant prescriptions before the index date were identified and duration of prescription was calculated (using data on quantity of tablets prescribed and numerical daily dosage)
- Oral corticosteroid exposure was defined as a 14 day course of high dosage (≥ 20 mg per day) oral corticosteroids in the month before the index date
- For body mass index (BMI), alcohol and smoking status, data were derived from medical Read codes and data from the additional details file. Read codes classifying patients by BMI category are very rarely recorded, and therefore were not used
- Where patients had multiple recordings, the nearest status in the period 1 year before to 1 month after index was taken (best); if not available, then the nearest status in the period 1 month after to 1 year after index was taken (second best); if not available, then the nearest status 1 year prior to index was taken (third best); if not available, then nearest status 1 year after index was taken (least best)

.

## Appendix 2

### Odds ratio for the association between length of most recent statin exposure and herpes zoster, stratified by time since last exposure

**Table:**

Statin use	Odds ratio (95% CI)		
	Model 1	Model 2	Model 3
Never	1·00	1·00	1·00
Current			
≤ 12 months	1·22 (1·19–1·25)	1·18 (1·15–1·21)	1·15 (1·12–1·18)
> 12 months	1·21 (1·19–1·24)	1·17 (1·15–1·20)	1·14 (1·11–1·17)
< 12 months since stopping statins			
≤ 12 months	1·13 (1·08–1·19)	1·10 (1·05–1·16)	1·07 (1·02–1·13)
> 12 months	1·23 (1·12–1·35)	1·20 (1·09–1·32)	1·15 (1·04–1·27)
12–36 months since stopping statins			
≤ 12 months	1·18 (1·11–1·25)	1·15 (1·08–1·23)	1·12 (1·05–1·19)
> 12 months	1·10 (0·96–1·26)	1·11 (0·96–1·27)	1·07 (0·93–1·23)
> 36 months since stopping statins			
≤ 12 months	1·12 (1·05–1·20)	1·08 (1·01–1·16)	1·05 (0·97–1·13)
> 12 months	1·20 (1·00–1·43)	1·20 (0·99–1·45)	1·16 (0·96–1·41)

CI, confidence interval. Model 1: unadjusted model. Model 2: unadjusted model, restricted to patients who had no missing data in all descriptive variables. Model 3: adjusted for body mass index category, smoking status, alcohol use, cardiovascular disease, HIV, lymphoma, leukaemia, myeloma, haematopoietic stem cell transplantation, other immunosuppressive therapy, other unspecified cellular immune deficiencies, oral corticosteroids, rheumatoid arthritis, systemic lupus erythematosus, chronic obstructive pulmonary disease, asthma, chronic kidney disease, depression and diabetes.

## Appendix 3

### Adjusted odds ratios (ORs) for association dosage of last statin prescription and herpes zoster, stratified by time since last statin prescription

The reference category for all estimations is patients who have never been prescribed a statin. All odds ratios are adjusted for body mass index category, smoking status, alcohol use, cardiovascular disease, HIV, lymphoma, leukaemia, myeloma, haematopoietic stem cell transplantation, other immunosuppressive therapy, other unspecified cellular immune deficiencies, oral corticosteroids, rheumatoid arthritis, systemic lupus erythematosus, chronic obstructive pulmonary disease, asthma, chronic kidney disease, depression and diabetes.

## Appendix 4

### Odds ratio for the association between ever having used a statin and herpes zoster, stratified by age

**Table:**

Statin use	Odds ratio (95% CI)		
	Model 1	Model 2	Model 3
Never	1·00	1·00	1·00
Ever			
< 70 years	1·21 (1·18–1·23)	1·17 (1·15–1·20)	1·12 (1·09–1·15)
≥ 70 years	1·20 (1·17–1·22)	1·16 (1·13–1·19)	1·13 (1·10–1·17)

CI, confidence interval. Model 1: unadjusted model. Model 2: unadjusted model, restricted to patients who had no missing data in all descriptive variables. Model 3: adjusted for body mass index category, smoking status, alcohol use, cardiovascular disease, HIV, lymphoma, leukaemia, myeloma, haematopoietic stem cell transplantation, other immunosuppressive therapy, other unspecified cellular immune deficiencies, oral corticosteroids, rheumatoid arthritis, systemic lupus erythematosus, chronic obstructive pulmonary disease, asthma, chronic kidney disease, depression and diabetes.

## Appendix 5

### Odds ratios for the association between both ever having been exposed to a statin plus time since last statin and herpes zoster

**Table:**

Statin use	Odds ratio (95% CI)		
	Model 1	Model 2	Model 3
Never	1·00	1·00	1·00
Ever	1·20 (1·18–1·22)	1·17 (1·15–1·19)	1·13 (1·11–1·15)
Current	1·21 (1·19–1·23)	1·18 (1·15–1·20)	1·14 (1·12–1·17)
≤ 3 months since stopping statins	1·16 (1·09–1·23)	1·13 (1·07–1·21)	1·09 (1·02–1·16)
> 3–12 months since stopping statins	1·15 (1·08–1·22)	1·11 (1·05–1·19)	1·08 (1·01–1·15)
> 12–36 months since stopping statins	1·17 (1·11–1·23)	1·15 (1·08–1·21)	1·11 (1·05–1·18)
> 36 months since stopping statins	1·13 (1·06–1·21)	1·09 (1·02–1·17)	1·06 (0·99–1·14)

CI, confidence interval. Model 1: unadjusted model. Model 2: unadjusted model, restricted to patients who had no missing data in all descriptive variables. Model 3: adjusted for body mass index category, smoking status, alcohol use, cardiovascular disease, HIV, lymphoma, leukaemia, myeloma, haematopoietic stem cell transplantation, other immunosuppressive therapy, other unspecified cellular immune deficiencies, oral corticosteroids, rheumatoid arthritis, systemic lupus erythematosus, chronic obstructive pulmonary disease, asthma, chronic kidney disease, depression and diabetes.
